# One Fell Swoop: Septic Muscle Embolism and Central Venous Catheter Infection Imaged with [^18^F] Fluorodeoxyglucose Positron Emission Tomography/Computed Tomography

**DOI:** 10.3390/diagnostics14020180

**Published:** 2024-01-14

**Authors:** Luca Filippi, Annamaria Lacanfora, Francesco Garaci

**Affiliations:** 1 Nuclear Medicine Unit, Department of Oncohaematology, Fondazione PTV, Policlinico Tor Vergata University Hospital, Viale Oxford 81, 00133 Rome, Italy; annamaria.lacanfora@ptvonline.it; 2 Department of Diagnostic Imaging, Molecular Imaging, University Hospital Tor Vergata, 00173 Rome, Italy; francesco.garaci@uniroma2.it

**Keywords:** positron emission computed tomography, infection, hybrid imaging, infective endocarditis

## Abstract

We describe the case of a 43-year-old female with hereditary hemochromatosis, previously without cardiac issues, who presented with a severe fever (>40 to 41 °C) to our hospital. Initial assessments, including transthoracic echocardiography, showed no typical signs of infective endocarditis. A contrast-enhanced CT scan revealed a hypodense area in the right subscapular muscle, alongside pleural thicknesses. Due to the critical condition, a central venous catheter (CVC) was implanted for immediate intravenous treatment. Subsequent blood cultures, positive for *Staphylococcus aureus*, and transesophageal echocardiography led to a diagnosis of multivalvular infective endocarditis (MIE). Subsequently, the patient underwent positron emission tomography/computed tomography (PET/CT) with [^18^F]Fluorodeoxyglucose ([^18^F]FDG), which detected increased tracer incorporation in the muscle lesion, CVC, and pleural thicknesses. The final diagnosis was CVC infection and septic embolism to the subscapular muscle in a patient with pleuritis. This case showcases the critical role of [^18^F]FDG PET/CT as whole-body imaging modality in diagnosing and managing complex infective cases.

**Figure 1:**
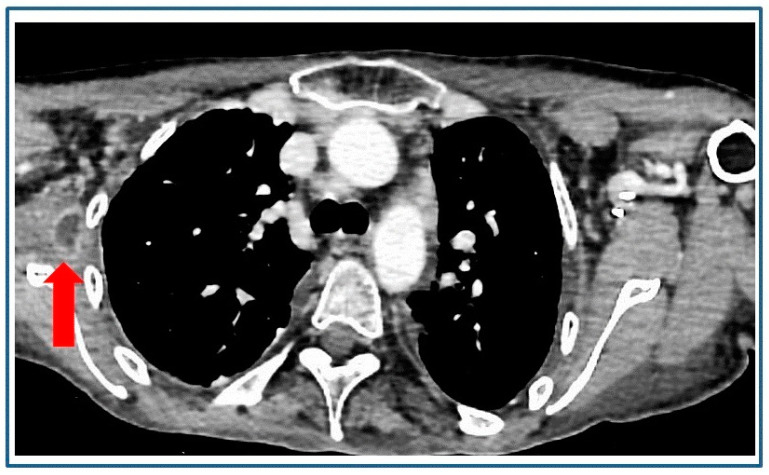
A 43-year-old female patient with hereditary hemochromatosis, submitted to regular check-up visits and without previous cardiac history, presented with a high fever level (>40 to 41 °C) and was in severely debilitating conditions. At admission, blood was drawn for standard hematologic tests, blood chemistries, and blood culture. Transthoracic echocardiography was also performed, revealing no evidence of typical patterns for infective endocarditis. Subsequently, a contrast-enhanced multislice computed tomography (ce-CT) scan detected a focal hypodense area with “rim enhancement” in the right subscapular muscle (red arrow), the clinical attribution of which remained uncertain, coupled with some pleural thicknesses. Due to the life-threatening nature of the situation and concerns about achieving adequate antibiotic concentrations, a central venous catheter (CVC) was implanted to promptly initiate immediate treatment with intravenous fluids and broad-spectrum antibiotics. After 72 h, patient’s overall conditions improved, with a fever decrease, and this allowed for the execution of further clinical investigations. Blood culture came back positive for *Staphylococcus aureus (S. aureus),* and transesophageal echocardiography findings led to a diagnosis of multivalvular infective endocarditis (MIE) due to mitral-aortic involvement.

**Figure 2:**
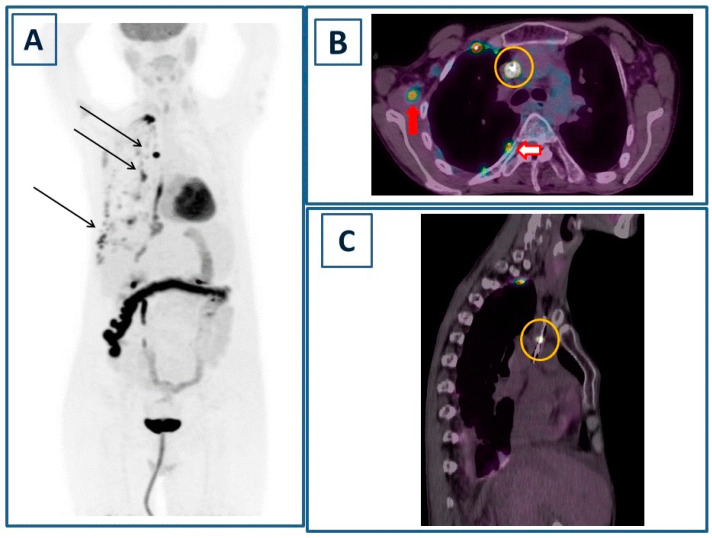
In order to better define the muscle lesion detected on ce-CT, positron emission tomography/computed tomography (PET/CT) was performed 60 min after the injection of 3.7 MBq/kg of [^18^F]Fluorodeoxyglucose ([^18^F]FDG). Maximum Intensity Projection (**A**) showed multiple areas of focal-increased tracer incorporation in the right hemithorax (black arrows), as well as non-specific uptake in the transverse colon and diffusely decreased liver uptake (mean standardized uptake value/SUVmean 1.69). The corresponding fused PET/CT axial image (**B**) showed focal tracer incorporation within the muscle lesion (red arrow, maximum standardized uptake value/SUVmax 3.6) and in correspondence to the CVC (yellow circle, SUVmax 32.6). Additionally, increased [^18^F]FDG consumption was observed due to multiple focal pleural thicknesses (red bordered arrows, SUVmax 8.4). Oblique sagittal projection (**C**) localized the tracer uptake in the intracaval portion of the CVC well (yellow circle). Of note, there were no identified foci of abnormal uptake at the level of the heart valves, probably related to the small size of the vegetations and the suboptimal preparation of the patient, resulting in an increased baseline cardiac activity. PET/CT findings were consistent with CVC infection and septic embolism to the right subscapular muscle in a patient with pleuritis. A PET/CT-guided biopsy of the muscle lesion, carried out to confirm the diagnosis, revealed necroinflammatory tissue with neutrophils. CVC was promptly removed and subjected to a culture test, which yielded a positive result for *S. aureus*. Multivalvular infective endocarditis (MIE) is a rare clinical condition with a prognosis that, despite advances in diagnosis and therapy, remains grim [1]. Septic embolisms are infrequent but are linked to elevated mortality and morbidity [2,3]. In these situations, imaging plays a crucial role in promptly detecting septic foci and monitoring the response to therapy. Nuclear medicine offers a diverse array of imaging techniques for the diagnosis and surveillance of infective endocarditis [4]. Scintigraphy using radiolabeled leukocytes for white blood cell (WBC) imaging has found extensive application in clinical practice. However, despite its high specificity, WBC scintigraphy is associated with certain limitations. These include the complex labeling procedure involving patients’ autologous leukocytes and the necessity for a multi-time-point acquisition protocol, encompassing early (1 h), delayed (4 h), and late (24 h) scans to capture the entire cell migration process [5]. Moreover, planar scans exhibit suboptimal spatial resolution, necessitating the use of hybrid single-photon computed tomography/computed tomography (SPECT/CT) to enhance diagnostic accuracy [6,7]. In recent years, [^18^F]FDG PET/CT has emerged as a prominent imaging modality for diagnosing infective endocarditis, as well as infections related to CVCs and implantable cardiac devices. This is attributed to the tracer’s ability to accumulate in activated leukocytes [8]. In comparison to SPECT/CT, PET/CT offers numerous advantages, including superior spatial resolution, an extended field of view (traditionally spanning from the skull base to proximal thighs), a streamlined one-day protocol, and the ability to achieve accurate quantification. Notably, PET/CT proves particularly valuable in detecting extracardiac infectious sites resulting from septic embolisms. While pulmonary septic embolisms, identified as focal points of increased tracer incorporation, have been extensively reported, the documentation of muscle septic embolisms through PET/CT remains limited [9,10,11]. Another important consideration pertains to the diffusely reduced uptake of [^18^F]FDG observed in the liver of our patient. In this context, a previously published retrospective study conducted on a substantial cohort (*n* = 1487) of cancer patients demonstrated that diminished liver uptake could be linked to poor nutritional status, anemia, impaired liver function, and systemic inflammation. Although the case we are describing is non-oncological, all the aforementioned conditions were present, suggesting a plausible explanation for the observed low hepatic [^18^F]FDG uptake [12]. In conclusion, our case underscores the significance of [^18^F]FDG PET/CT in this context and sets the stage for future investigations into the recently introduced Long Axial Field-of-View (LAFOV) scanners. These PET/CT scanners boast superior sensitivity, enabling low-dose and rapid protocols. This advancement holds promise for further enhancing our understanding and diagnostic capabilities in the field [13,14].

## Data Availability

The data presented in this article are available on request from the corresponding author.
